# Modelling and predicting online vaccination views using bow-tie decomposition

**DOI:** 10.1098/rsos.231792

**Published:** 2024-02-21

**Authors:** Yueting Han, Marya Bazzi, Paolo Turrini

**Affiliations:** ^1^ MathSys CDT, University of Warwick, Coventry, UK; ^2^ Mathematics Institute, University of Warwick, Coventry, UK; ^3^ Department of Computer Science, University of Warwick, Coventry, UK; ^4^ The Alan Turing Institute, London, UK

**Keywords:** computational social science, social networks, data analysis, opinion dynamics, social psychology

## Abstract

Social media has become increasingly important in shaping public vaccination views, especially since the COVID-19 outbreak. This paper uses bow-tie structure to analyse a temporal dataset of directed online social networks that represent the information exchange among anti-vaccination, pro-vaccination and neutral Facebook pages. Bow-tie structure decomposes a network into seven components, with two components, strongly connected component (SCC) and out-periphery component (OUT), emphasized in this paper: SCC is the largest strongly connected component, acting as an ‘information magnifier’, and OUT contains all nodes with a directed path from a node in SCC, acting as an ‘information creator’. We consistently observe statistically significant bow-tie structures with different dominant components for each vaccination group over time. In particular, the anti-vaccination group has a large OUT, and the pro-vaccination group has a large SCC. We further investigate changes in opinions over time, as measured by fan count variations, using agent-based simulations and machine learning models. Across both methods, accounting for bow-tie decomposition better reflects information flow differences among vaccination groups and improves our opinion dynamics prediction results. The modelling frameworks we consider can be applied to any multi-stance temporal network and could form a basis for exploring opinion dynamics using bow-tie structure in a wide range of applications.

## 1. Introduction

Vaccination campaigns have drawn long-standing public attention [1,2], particularly since the outbreak of the COVID-19 pandemic [3–6]. Given the significant impact of online social media platforms as sources of information, a number of studies have emphasized their effect on vaccination views in public opinion [7–12]. Recent studies have highlighted the significance of the information ‘creator–receiver’ dynamics in online vaccination campaigns: some researchers have found that vaccination opponents tend to produce a higher volume of information than vaccination supporters [12–15]; several studies have observed that most (mis)information is created by a minority of users (which should not be assumed to be representative of a majority) and that information roles tend to remain relatively stable over time [15,16].

Building on these previous studies, this paper explores online behavioural differences among vaccination groups, namely vaccination supporters, opponents and neutrals. Instead of simply dividing online users into two categories (i.e. ‘creators’ and ‘receivers’) based on the volume of messages they create or receive, this paper explores a more nuanced division of roles each user might play in online information flow using a network structure called ‘bow-tie structure’. It was recently explored in the context of online debates [17], and we introduce it next.

### 1.1. Bow-tie structure

**Figure 1.**
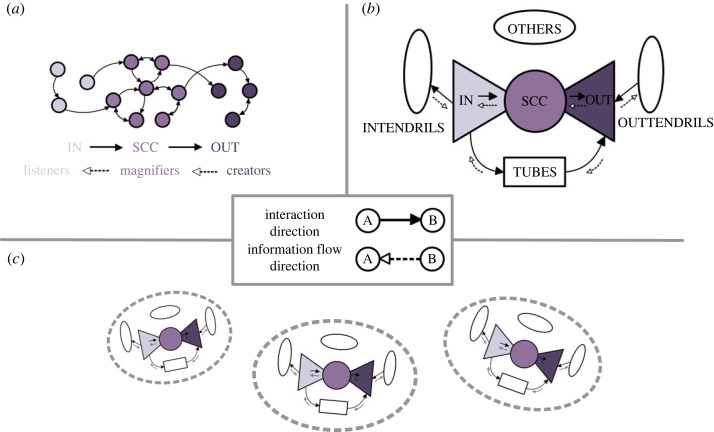
Bow-tie structures in online social networks. The arrows highlight that in this paper, an edge from node A to B in online social networks (solid arrow) represents an interaction from page A to B (e.g. page A recommends page B to its members), while the direction of information flow (dashed arrow) goes in the opposite direction (e.g. content about page B is presented or ‘flows’ to page A). (*a*) Primitive bow-tie structure. This panel illustrates a bow-tie structure that divides a toy example network into three components: SCC, IN and OUT. This decomposition establishes pairwise relations between these components, assigning distinct roles to each in terms of information flow: IN, ‘listeners’; SCC, ‘magnifiers’; OUT, ‘creators’. (*b*) Extended bow-tie structure. This panel expands on the bow-tie structure in panel *a* by introducing additional components: TUBES, INTENDRILS, OUTTENDRILS and OTHERS. In this structure, IN not only ‘listens’ to SCC but also to INTENDRILS and TUBES, while OUT not only ‘creates’ information that is delivered to SCC but also to TUBES and OUTTENDRILS. (*c*) Recursive bow-tie structure. This panel displays an example of a recursive bow-tie structure, where the entire graph is partitioned into subgraphs, and bow-tie decomposition is applied to each of them. Note that edges across partitioned subgraphs are disregarded in this case.

Bow-tie structure was introduced by Broder *et al.* [18] in 2000 as a type of network structure that encodes the connectivity of the World Wide Web (WWW), with nodes representing pages and edges representing hyperlinks. The primitive form of bow-tie structure divides a directed network into four components: the largest strongly connected component (SCC), the in-periphery component (IN) which includes all nodes with a directed path to a node in SCC, the out-periphery component (OUT) which comprises all nodes with a directed path from a node in SCC and the other sets (OTHERS) for all remaining nodes. The first three components of bow-tie structure (figure 1*a*) are shaped like a bow-tie, with SCC acting as the knot and IN and OUT components as the fans. Bow-tie structure was later refined by Yang *et al.* [19] through the introduction of TUBES, INTENDRILS and OUTTENDRILS, as shown in figure 1*b*. Yang *et al.* also proved that any directed graph can be decomposed into a bow-tie structure.

Online social media can be viewed through a similar lens to that of the WWW, where pages are run by social media users and directed interactions exist between pages, such as thumbs-up, reposts and following [20]. In this paper, we define an edge from node A to B in an online social network as an interaction from page A to page B (e.g. page A recommends page B). Such an interaction is often triggered by page B presenting some content of interest to page A.

In the light of this, bow-tie structure can provide useful insight into interpreting the various ‘role’ of pages in online information flow [17,21,22]. Pages in SCC are usually active in two-way interactions (e.g. sharing strong communicative interests), and thus may be regarded as information ‘magnifiers’. OUT pages are often passively interacted with by SCC, INTENDRILS and TUBES pages but seldom engage in directed interactions with these pages. This suggests a high tendency of OUT pages to present their content to the public, behaving as information ‘creators’. IN pages generally act in the opposite way, serving as information ‘listeners’ to pages in SCC, OUTTENDRILS and TUBES. OTHERS usually contains pages with sparse interactions with most pages in other bow-tie components, either proactively or passively. For finer-scale analysis of such information flow, recursive bow-tie structures have been explored by considering bow-tie structures of subgraphs, in contrast to the entire network [17,23] (see an illustration in figure 1*c*). Subgraphs are often extracted through the application of computational methods [17,23], such as community detection where ‘densely connected’ users are grouped together [23]. Manual examination may also be employed to ensure users sharing specific discussion topics are appropriately grouped [17].

To investigate bow-tie structure in online vaccination campaigns, we study a real-world temporal dataset about an online vaccination campaign, which is publicly available and was previously analysed in 2020 by Johnson *et al.* [13]. It describes two snapshots of online recommendations between Facebook pages in February and October 2019 (before the COVID-19 outbreak), with each page manually checked and assigned a vaccination stance of ‘anti’, ‘pro’ or ‘neutral’.^1^ The dataset was originally analysed based on page-level interactions, the number of members who subscribe to each page (i.e. the ‘fan size’), narratives (e.g. safety concerns and conspiracy theories) and geography. Information on geography and narratives was not made public by the authors and is currently not available. Data on vaccination stance, time stamps and fan size allow us to investigate the following important questions about bow-tie structures: (i) explanatory power, i.e. whether the stance on vaccination is associated with a different bow-tie structure, and how to explain any existing differences; (ii) temporal stability, i.e. whether bow-tie structures remain stable through time; (iii) predictability, i.e. whether bow-tie structures can help predict fan size variations, serving as a reflection of the dynamic nature of online vaccination views.^2^

### 1.2. Contribution

This paper uses bow-tie decomposition to analyse and predict online vaccination views. We build on Mattei *et al.*’s contribution [17], which identifies different recursive bow-tie structures in online social networks, and apply this idea to the online vaccination views dataset from Johnson *et al.* [13,14]. To our knowledge, there is very limited prior research applying bow-tie structure to investigate and predict the spread of opinion dynamics in social media networks. Additionally, while Johnson *et al.* [13] provide an opinion dynamics prediction model on the same dataset that relies solely on page fan counts and disregards network structures, our study incorporates bow-tie structure into the modelling framework.

The contribution of this paper is twofold: firstly, we find that online vaccination groups (i.e. pages holding anti-vaccination, pro-vaccination and neutral viewpoints) exhibit different bow-tie structures, which we interpret in the light of information flow roles. Secondly, using agent-based epidemic simulations and machine learning models, we explore how these structures reflect information flow differences among vaccination groups and their potential to predict online vaccination view dynamics as quantified by fan size page variation. The modelling frameworks we use are general and could form a basis for exploring opinion dynamics using bow-tie structure in a wide range of applications.

### 1.3. Paper structure

This paper is organized as follows. In §2, we describe the online recommendation dataset about vaccination views. Section 3 outlines the methodology of bow-tie decomposition in this paper. In §4, we present our findings by first detecting and interpreting bow-tie structures in this dataset, and secondly using this structure in agent-based and machine learning models to predict dynamics in online vaccination views. Finally, §5 summarizes our main results and discusses directions of future work.

## 2. Data description

**Figure 2.**
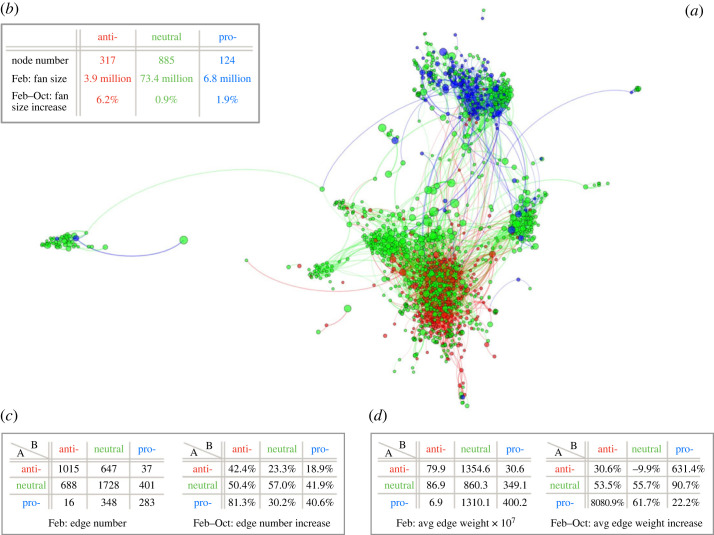
Online recommendation networks about vaccination views. (*a*) February network. This is a snapshot of the largest weakly connected subgraph in February 2019, reproduced from Johnson *et al.* [13]. It includes over 94% of nodes and 99% of edges from the entire network. Each node represents a page. Its node colour depicts page polarity: red for anti, green for neutral, and blue for pro. Its node size is proportional to its page fan size. The node layout follows ForceAtlas2 in Gephi. The edge colour follows the colour of its source node (where the edge starts from). (*b*) Node-level data. It describes the total number of nodes and the total fan size for each vaccination group. By observation, the neutral vaccination group dominates with the largest number of pages and fans. The pro-vaccination group has fewer pages but a stronger fan base than the anti-vaccination group, mainly due to three pages with over a million fans. The anti-vaccination group has no pages with over a million fans in February and October but experienced the largest percentage increase in fans from February to October. (*c*,*d*) Edge-level data. It describes the edge number (panel *c*) and the average edge weight (panel *d*) within and across vaccination groups. Every edge is directed from A to B (i.e. A recommends B). It can be observed that the direction and weight of recommendations are important. Despite that, there are a larger number of edges within vaccination groups than across vaccination groups (except for pro- pages), and the highest edge weights flow from anti- to neutral and pro- to neutral groups (possibly due to the neutral group’s high activity in interacting with both groups). Additionally, the anti- and pro-vaccination groups had minor interaction in February but interestingly experienced drastic increases in both edge number and average weight from February to October.

The dataset from Johnson *et al.* [13] consists of two snapshots of online competition between different vaccination views in February and October 2019, involving nearly 100 million users on Facebook across countries, continents and languages. It can be represented by two directed networks corresponding to February and October. The February network is illustrated in figure 2*a*. The number of nodes is the same in February and October, given by 1326 in total (see further data in figure 2*b*). The number of edges in February is 5163, and in October is 7484 (see further data in figure 2*c*). The original data was in .pdf form, we pre-process it and make it easily accessible in a variety of analysis-ready formats on GitHub: https://github.com/YuetingH/BT_Vaccination_Views.

Details are explained below:

— *Node.* Each node represents a public Facebook page that discusses vaccination topics. It is attributed with fan size, that is, the number of members who subscribe to the Facebook page, along with the other attribute polarity including anti-vaccination, pro-vaccination and neutral. Here, neutral pages ‘focus around vaccines or another topic (e.g. a school parent association that has become linked to the vaccine debate but for which the stance is still undecided)’, as introduced by Johnson *et al.* [13]. Whereas its polarity remains the same for February and October snapshots, its fan size can either increase or decrease.
  Remarks. (i) For consistency, a red, blue, or green node will always represent the page in the anti-, pro-vaccination and neutral group when it comes to analysing this dataset. (ii) It is allowed for a page to have no fans (this is the case for a total of 4 pages in February and 10 pages in October).
— *Edge.* A directed edge from node A to B means page A recommends B to all its members at the page level, as opposed to a page member simply mentioning another page. The number of times a page is recommended and the exact timestamp of the recommendation is not recorded in the dataset. Rather, an edge from A to B is present in a given monthly snapshot if page B was recommended to all members of A at some point earlier or within the month. In that sense, the network represents the cumulative recommendations over time, with edges in February also appearing in October, but not the other way around. Note that, despite the cumulative nature of the edges, the differences between the two snapshots are evident and visually shown in [13] figure 2*a*.
  Remarks. Both ‘two-way recommendation’ and ‘self-recommendation’ are allowed. Two-way recommendation means two pages recommend each other (involving 8.5% of all recommendations in February and 10.2% in October). Self-recommendation, where a page recommends itself to all of its fans (e.g. to increase engagement), is rare in our dataset (less than 0.2% in both February and October).

During preprocessing, we define an *edge weight* to quantify the significance of each recommendation (figure 2*d*). It is obtained by the product of both ends’ fan size. This edge weight choice builds on the intuition that more fans may be recruited if pages on both ends have larger fan sizes. On the one hand, being recommended to another page with a larger fan size will probably attract more fans. On the other hand, a recommended page with a larger fan size is potentially more influential to other pages; thus, more fans may be recruited accordingly. This weight choice is also mentioned as a ‘product kernel’ by Johnson *et al.* in their prediction model [13], which was previously shown to be useful in Palla *et al.* [24]. Although bow-tie structure disregards edge weight, community detection involved in recursive bow-tie structure can incorporate this factor, so as to produce a better partition of the network. See details in §3.2.

## 3. Methodology

We focus on a particular network type throughout this paper, aligned with our dataset: directed weighted networks with self-loops and without multi-edges. Some preliminary graph definitions are listed below.

— A *network* *G* = (*V*, *A*) consists of a set of nodes *V* and a weighted adjacency matrix *A* = (*A*_*u*,*v*_)_*u*,*v*∈*V*_. *A*_*u*,v_ = *w* > 0 if there is an edge from node *u* to *v* with weight *w* and *A*_*u*,*v*_ = 0 otherwise.
— A *path* from *u* ∈ *V* to *v* ∈ *V* is defined as a succession of nodes (*n*_0_, *n*_1_, …, *n*_*k*_), where *k* is a non-negative integer, *n*_0_ = *u*, *n*_*k*_ = *v*, and for any *i* = 1, …, *k*, *n*_*i*_ ∈ *V* are distinct satisfying $A_{n_{i-1},n_{i}}>0$. As a special case, every node *u* ∈ *V* is considered to have a path (*n*_0_ = *u*) to itself.
— Let *u*, *v* ∈ *V* be nodes and *T*⊆ *V* be a subset of nodes. A node *v* is said to be *reachable* from *u* if a path exists from *u* to *v*. As an extension, *u* is said to be reachable from *T* if there exists at least a *w* ∈ *T* such that *u* is reachable from *w*. *T* is said to be reachable from *u* if there exists at least a *w* ∈ *T* such that *w* is reachable from *u*.
— A *subgraph* *G*′ = (*V*′, *A*′) of a graph *G* = (*V*, *A*) is a graph such that *V*′⊆ *V* and *A*′ satisfies *A*′_*u*,*v*_ = *A*_*u*,*v*_ for any *u*, *v* ∈ *V*′.
— A *strongly connected component* of a graph *G* is a subgraph of *G* where there exists a path from every node to every other node.
— A *hard partition* of a graph *G* is a division of the set of nodes *V* into *k* non-overlapping sets *C*_*i*_, *i* = 1, 2, …, *k*, such that $\overset{k}{\underset{i=1}{⋃}}C_{i}=V$ and $C_{i}\cap C_{j}=\emptyset$ for any *i* ≠ *j*. One can also consider soft partitions, where sets can overlap, but this is beyond the scope of this paper. We refer to a ‘hard partition’ as a ‘partition’ throughout the paper.

### 3.1. Bow-tie structure

In this paper, we adopt the bow-tie structure definition and detection algorithm presented by Yang *et al.* [19], also used in Mattei *et al.* [17].

**Definition.**

*Assume* *S* *is the largest strongly connected component of* *G*. *Bow-tie structure of* *G* *consists of the following sets of nodes*:

$$\text{SCC}=S,\;\text{IN}=\{v\in (V-S)\;|\;S\;\text{is reachable from}\;v\}{,}^{3}\;\;\text{OUT}=\{v\in (V-S)\;|\;v\;\text{is reachable from}\;S\}$$

^[Fn FN3]^

TUBES={v∈(V−S−IN−OUT) | v is reachable from IN and OUT is reachable from v}

INTENDRILS={v∈(V−S) | v is reachable from IN and OUT is not reachable from v}

OUTTENDRILS={v∈(V−S) | v is not reachable from IN and OUT is reachable from v}

OTHERS=V−S−IN−OUT−TUBES−INTENDRILS−OUTTENDRILS

Yang *et al.* [19] also proved that the sets of bow-tie components above are mutually disjoint and thus form a partition of the nodes. In other words, any directed graph can be decomposed into a bow-tie structure.

*Algorithm.* The detection of the largest strongly connected component is a well-established process in the field of graph theory, with early works from [25,26]. The algorithm for obtaining the remaining bow-tie components is outlined by Yang *et al.* [19]. The entire algorithm for detecting bow-tie structure has been implemented in code on GitHub, and we employ the same code for our analysis.

### 3.2. Recursive bow-tie structure

#### Background

Bow-tie structure of an entire network is largely dependent on the generation of its edges, which, however, often exhibit some amount of randomness in online social networks [14,27]. For instance, users may randomly interact with recommended strangers [27,28]. Social bots, creating fake accounts and spreading spam, also contribute to this randomness [28,29]. This has motivated researchers to move beyond the potential randomness of edges and delve deeper into the analysis of bow-tie structure on a finer scale, also referred to as ‘recursive bow-tie structure’.

Recursive bow-tie structure has been explored by considering partitioned subgraphs or different choices of SCC. The first considers bow-tie decomposition on partitioned subgraphs while ignoring inter-subgraph edges. This approach is grounded on the empirical observation that online social networks usually exhibit community structures, where nodes within a set are densely connected and inter-set connections are relatively sparse [23,28,30]. These communities are often composed of users who share common communicative goals, referred to as ‘discursive communities’ by Mattei *et al.* [17]. For instance, in their research, bow-tie decomposition is applied to communities including left/right politicians and official accounts of governments and media (e.g. newspapers, TV channels and journalists) examined through metadata. Another example from Fujita *et al.* [23] employs a computational technique to perform bow-tie analysis on densely connected communities. Bow-tie role assignments produced by this type of recursive bow-tie structure are interpretable, as they reflect the local roles of nodes within each subgraph. The second type of recursive bow-tie structure considers other strongly connected components rather than the largest one [19,31,32]. This second method, while theoretically feasible, is often less interpretable as it may assign multiple roles to the same nodes.

*Algorithm.* This paper focuses solely on the first approach (see its implementation in algorithm 1), which considers bow-tie structures of partitioned discursive communities as described by Mattei *et al.* [17]. Associated with our online vaccination view dataset, we interpret the discursive communities in two ways: (1) vaccination groups (anti-, pro-, neutral) and (2) densely connected communities detected by computational techniques, where the number or size of communities is not fixed. As a result, two kinds of decomposition will be implemented for each network snapshot, enabling intra (i.e. approach (1)) and inter (i.e. approach (2)) vaccination groups’ bow-tie analysis over time.

Note that while subgraphs of vaccination groups can be easily extracted based on the metadata from Johnson *et al.* [13], community detection typically requires the use of computational heuristics, with multiple choices available [33–37]. For the purposes of this paper, we use a widely used community detection method known as Infomap. This approach leverages Shannon Entropy and random walks with edge weights for community detection [35]. It introduces some stochasticity to the identified partition, but our analysis indicates that it has a minor influence on our results. Infomap may also detect small communities with fewer than five nodes, but these are less meaningful in our context. To address this, we label nodes in such communities as ‘UNASSIGNED’. We provide further details in the electronic supplementary material to explain why we choose Infomap over modularity maximization [33] (another popular community detection method) for our specific problem, and examine the stochasticity of Infomap in our dataset.

**Algorithm 1.** Recursive bow-tie detection.

**Input:** graph *G*, partition *C* = {*C*_1_, …, *C*_*k*_}

**Output:** SCC^r^, IN^r^, OUT^r^, TUBES^r^, INTENDRILS^r^, OUTTENDRILS^r^, OTHERS^r^

1: For *i* = 1, …, *k*, construct a subgraph *G*_*i*_ = (*C*_*i*_, *A*_*i*_) of the graph *G*.
2: For each subgraph *G*_*i*_, acquire its bow-tie structure SCC_*i*_, IN_*i*_, OUT_*i*_, TUBES_*i*_, INTENDRILS_*i*_, OUTTENDRILS_*i*_, OTHERS_*i*_.
3: Obtain ${\text{SCC}}^{r}=\overset{k}{\underset{i=1}{⋃}}{\text{SCC}}_{i}$. Repeat this step to obtain other bow-tie components IN^r^, OUT^r^, TUBES^r^, INTENDRILS^r^, OUTTENDRILS^r^, OTHERS^r^.

## 4. Results

Our study focuses on two aspects: the detection of bow-tie structures in recommendation-based online social networks, and the analysis of these structures to enhance the prediction of opinion dynamics (modelled as page fan size over time). Sections 4.1 and 4.2 will discuss these two aspects, respectively. Section 4.1 explores whether the stance on vaccination is associated with different bow-tie structures, examines the stability of such structures over time, and assesses how one can interpret these observations. Section 4.2 uses and compares two approaches to predict page fan count variation: (i) supervised machine learning and (ii) mechanistic simulation via agent-based epidemic models on information cascade.

As mentioned in §3.2, we consider bow-tie structures of ‘discursive communities’ in the February–October 2019 network snapshots following Mattei *et al.* [17], where bow-tie structure is identified in subgraphs of the network (figure 1*c*). We characterize discursive communities in two ways that can yield complementary insights: (i) their view, in our case pro-, anti- and neutral-vaccination groups; (ii) their placement in densely connected sets within the network, as defined by community structure, which we detect using the commonly used flow-based method Infomap [35]. In this sense, two kinds of bow-tie decomposition are implemented for each network snapshot, enabling intra and inter vaccination groups bow-tie analysis. Each page is assigned a dual bow-tie role (different or not, such as SCC–SCC, SCC–OUT) at each timestamp. For clarity in our explanations, we refer to the first coarse-graining of discursive communities (i.e. when subgraphs are vaccination groups) as ‘within-group bow-tie structure’, and a page’s assigned role as its ‘within-group bow-tie role’. On the other hand, we term the second coarse-graining of discursive communities (i.e. when subgraphs are communities identified with Infomap) as ‘across-group bow-tie structure’, and a page’s assigned role as its ‘across-group bow-tie role’.

### 4.1. Bow-tie structure detection in the recommendation networks

**Figure 3.**
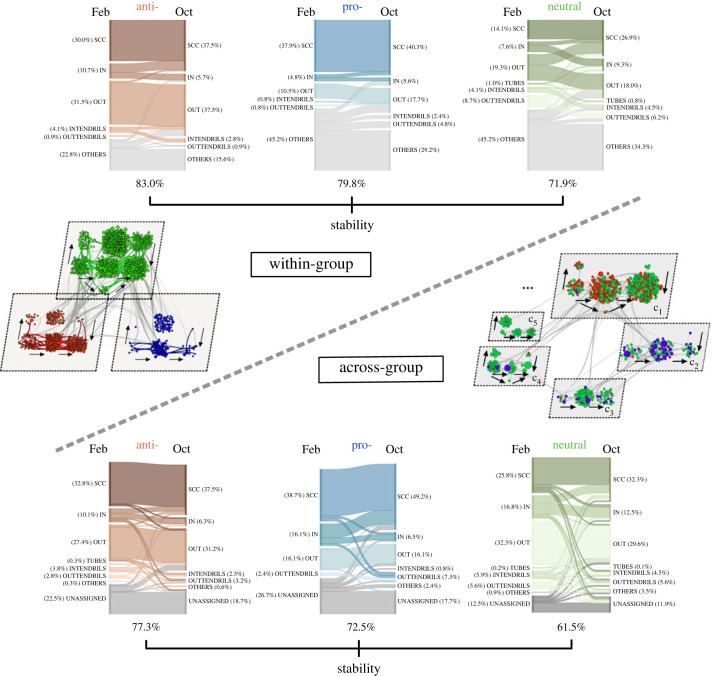
Within-group and across-group bow-tie structures in the February and October 2019 online recommendation-based networks. The figure displays within-group bow-tie structures in the upper part and across-group bow-tie structures in the lower part for networks at both timestamps. Each part includes an explanatory diagram of the decomposition scheme, where the February network is divided into subgraphs, and the bow-tie structure within each subgraph is revealed using an organized layout of node and arrow, maintaining consistency with figure 1*b*. Nodes are colour-coded by vaccination group and proportionally sized based on fan size, consistent with figure 2*a*. Note that the across-group diagram shows the largest five communities, labelled as *C*_*i*_, ranked by node counts, with larger communities having smaller indices, which collectively represent 49.4% of all pages. While all five largest communities are primarily composed of neutral pages, communities *C*_1_ and *C*_2_ stand out with nearly half of their pages anti- and pro-vaccination, respectively. Three Sankey diagrams in each part illustrate the bow-tie structures for pages with different vaccination views. Each diagram has two columns representing bow-tie roles for February and October, with the flow indicating role variations.^4^ The stability, indicated beneath each diagram, quantifies the percentage of pages that maintain the same bow-tie roles at both timestamps. Overall, these results indicate that the pro-vaccination group exhibits a large SCC in bow-tie decomposition for both choice of discursive communities, while the anti-vaccination group is comparatively dominated by OUT component. Moreover, these structures are stable over time. In contrast, the neutral group yields inconsistent bow-tie structures and exhibits less temporal stability.

Our results in figure 3 indicate that bow-tie structures associated with the pro-vaccination group consistently exhibit a large SCC in either way of bow-tie decomposition and at both February and October timestamps, whereas those associated with the anti-vaccination group have a comparatively large OUT component. By contrast, the neutral group demonstrates inconsistent results, with large OTHERS in within-group bow-tie decomposition, but a dissimilar pattern in across-group bow-tie decomposition with comparatively large main bow-tie components (i.e. SCC, OUT and IN). Furthermore, anti-vaccination and pro-vaccination pages display a higher temporal stability in their bow-tie structures than neutral pages when transitioning from February to October, with anti-vaccination pages being slightly more stable than pro-vaccination pages.

We offer some interpretations of the detected bow-tie structures. The large OUT component of the anti-vaccination group suggests a strong commitment to generating information. The pro-vaccination group’s large SCC component underscores its strong information dissemination capability. Neutral pages have limited interactions with the ‘mainstream’ (i.e. SCC, OUT, IN components) and are assigned the ‘OTHERS’ role in the within-group bow-tie structure. By contrast, their interactions with anti- and pro-vaccination pages are more likely to focus on vaccination topics, resulting in strong across-group bow-tie structures with relatively large SCC, OUT and IN components. Finally, in this dataset, pages in marginal components (i.e. OTHERS, INTENDRILS, OUTTENDRILS and TUBES) tend to transition towards main components (i.e. SCC, OUT and IN) over time. This shift may reflect increased integration into mainstream discussions, either through referencing SCC pages or being referenced by them.

Interestingly, while these observations are obtained using a different methodology (bow-tie structure), several are consistent with those discussed in Johnson *et al.* [13] using node metadata. For example, the authors in [13] mention that anti-vaccination groups generate a diverse range of narratives that blend topics such as ‘safety concerns’ and ‘conspiracy theories’. Furthermore, the pro-vaccination pages in the dataset are more centralized geographically than the anti-vaccination pages, which can facilitate reciprocal recommendations and amplify information dissemination. Neutral pages among themselves touch on a variety of topics (e.g. parenting and pets pages [13,14]) rather than mostly vaccination topics, which may help explain why they belong to ‘OTHER’ when discursive communities are associated with vaccination stances.

We end this section with observations on the robustness of our results. Firstly, we find that the detected bow-tie structures are statistically significant through comparisons with appropriately generated random graphs. More details can be found in the electronic supplementary material. Secondly, the follow-up dataset with two additional snapshots (i.e. November 2019 and December 2020) yields recursive bow-tie analysis results that are generally consistent with our findings in the dataset presented previously. Again, see more details in the electronic supplementary material. Thirdly, we observe that the temporal stability of across-group bow-tie decomposition is overall lower than that of within-group decomposition. This is not surprising, in the sense that the higher and varying number of detected communities compared with the constant small number of vaccination groups may favour more variation between snapshots.

### 4.2. Experiments with detected bow-tie structure on opinion dynamics prediction

Next, we examine whether one can use the February 2019 recommendation network to help predict fan count disparities for each individual page between February and October 2019, as a way to gain insight into opinion dynamics during this period. These predictions are implemented using supervised machine learning models and agent-based susceptible-infected-recovered (SIR) models, with a specific emphasis on exploring whether bow-tie structure can improve these predictions.

It is important to note that, *a priori*, one might expect recommendations to be more strongly correlated with fan size increase, than with fan size decrease. In other words, pages that receive recommendations are potentially likely to witness an increase in their fan counts over a short time frame, while those not recommended may not necessarily experience a decline. We come back to this point when discussing our results.

Furthermore, we establish two assumptions below to centralize our research focus and enhance the interpretability of our results. (i) We exclusively perform predictions on anti- and pro-vaccination pages, excluding neutral pages (while not ignoring the interactions of anti- and pro-pages with neutral pages). This choice is motivated by the fact that vaccination topics may not constitute the primary focus of neutral pages, such as those centred around parenting and pets [13,14], and their fan count fluctuations may have a limited connection to recommendation networks concerning vaccination views. (ii) Instead of covering all bow-tie components, we narrow our analysis to three key bow-tie components: SCC, OUT and IN, while grouping the remaining components as ‘NA’. This decision is made based on the following reasons. Components SCC, OUT and IN offer clearer insights for interpreting the roles of bow-tie components in information propagation. For instance, IN pages ‘listen’ to SCC, INTENDRILS and TUBES pages (main listeners), while OUTTENDRILS pages ‘listen’ to OUT pages only (marginal listeners). In addition, our empirical observations above show that the page count in SCC and OUT components effectively distinguishes between anti-vaccination and pro-vaccination pages, whereas other components lack such clarity.

#### 4.2.1. Supervised machine learning

We begin by extracting categorical and numeric features of the anti- and pro-vaccination pages, including those related to bow-tie structure, from the February 2019 recommendation network snapshot, which potentially contribute to variations in page fan counts compared with October 2019 (table 1).

**Table 1.** Extracted Features from February 2019 Online Recommendation-based Network Snapshot. We study features of pro- and anti-vaccination pages in order to predict their fan count variations (i.e. *i* = *r*, *b*).

notation	description	type	remarks
*p*_*i*_	The polarity of page *i*, which is denoted as follows: ‘r’ for anti-, ‘b’ for pro- and ‘g’ for neutral^a^	categorical	In our ML models (i.e. LR, SVR and RFR), we use one-hot encoding to convert categorical features into a binary vector format.
*c*_*i*_	The Infomap community assignment of page *i*, encoded with integers ranging from 1 to 172 (i.e. a total of 172 distinct communities were detected)	categorical	
*W* − *BT*_*i*_	The within-group bow-tie role of page *i*, which can be SCC, IN, OUT and NA	categorical	
*A* − *BT*_*i*_	The across-group bow-tie role of page *i*, which can be SCC, IN, OUT and NA	categorical	
*f*_*i*_	The fan count of page *i*	numeric	Log transformation implemented to improve our ML model performance
$k_{i}^{\mathrm{in}}\;:=\sum_{\;j\in \{r,\;b,\;g\}}A_{\;ji}$ ^b^	The weighted in-degree of page *i* (recalling that edge weight is obtained by the product of fan counts at both ends), indicating the accumulated strength of recommendations made to page *i*	numeric	We find that neutral pages seldom reciprocate recommendations with both anti- and pro-vaccination pages. Instead, if neutral pages interact (i.e. recommend or are recommended) with non-neutral counterparts, a clear one-sided leaning emerges. Additionally, anti- (pro-) pages predominantly engage with similar-minded or neutral pages, rarely with pro- (anti-) ones. These trends are visually shown in the electronic supplementary material. Consequently, we do not delineate features indicating the proportion of inter vaccination group page interactions with specific vaccination groups, and we solely specify the features indicating the proportion of within vaccination group page interactions.
$k_{i}^{\mathrm{out}}\;:=\sum_{\;j\in \{r,\;b,\;g\}}A_{ij}$	The weighted out-degree of page *i*, indicating the accumulated strength of recommendations made by page *i*	numeric	
$k-PS_{i}^{\mathrm{in}}\;:=\frac{\sum_{\;j\in \{r,\;b,\;g\}}1_{\;p_{i}=p_{j}}A_{\;ji}}{\sum_{\;j\in \{r,\;b,\;g\}}A_{\;ji}}=\frac{\sum_{\;j\in \{r,\;b,\;g\}}1_{\;p_{i}=p_{j}}A_{\;ji}}{k_{i}^{\mathrm{in}}}$	The proportion of the recommendation strength from pages with the same polarity as page *i*, out of all the accumulated recommendations made to page *i*	numeric	
$k-PS_{i}^{\mathrm{out}}\;:=\frac{\sum_{\;j\in \{r,\;b,\;g\}}1_{\;p_{i}=p_{j}}A_{ij}}{\sum_{\;j\in \{r,\;b,\;g\}}A_{ij}}=\frac{\sum_{\;j\in \{r,\;b,\;g\}}1_{\;p_{i}=p_{j}}A_{ij}}{k_{i}^{\mathrm{out}}}$	The proportion of the recommendation strength to pages with the same polarity as page *i*, out of all the accumulated recommendations made from page *i*	numeric	
$k-CS_{i}^{\mathrm{in}}\;:=\frac{\sum_{\;j\in \{r,\;b,\;g\}}1_{c_{i}=c_{j}}A_{\;ji}}{\sum_{\;j\in \{r,\;b,\;g\}}A_{\;ji}}=\frac{\sum_{\;j\in \{r,\;b,\;g\}}1_{c_{i}=c_{j}}A_{\;ji}}{k_{i}^{\mathrm{in}}}$	The proportion of the recommendation strength from pages with the same Infomap community assignment as page *i*, out of all the accumulated recommendations made to page *i*	numeric	
$k-CS_{i}^{\mathrm{out}}\;:=\frac{\sum_{\;j\in \{r,\;b,\;g\}}1_{c_{i}=c_{j}}A_{ij}}{\sum_{\;j\in \{r,\;b,\;g\}}A_{ij}}=\frac{\sum_{\;j\in \{r,\;b,\;g\}}1_{c_{i}=c_{j}}A_{ij}}{k_{i}^{\mathrm{out}}}$	The proportion of the recommendation strength to pages with the same Infomap community assignment as page *i*, out of all the accumulated recommendations made from page *i*	numeric	
*PageRank*_*i*_	The pagerank of page *i*	numeric	Commonly employed features in research concerning the dynamics of online vaccination-related opinions (e.g. pagerank: [38–40]; betweenness: [38,41,42])
*Betweenness*_*i*_	The betweenness of page *i*	numeric	

^a^Recall that to distinguish pages from anti-vaccination, pro-vaccination and neutral groups, we have used a colour scheme of red, blue and green. Here we abbreviate them as ‘r’, ‘b’ and ‘g’, respectively.

^b^As shown in the beginning of §3, we define *A*_*ji*_ = *ω* > 0 if there is an edge from node *j* to *i* with weight *ω* (i.e. page *j* recommends page *i* with recommendation strength *ω*) and *A*_*j*,*i*_ = 0 otherwise.

To investigate whether our supervised ML models are more feasible in predicting fan count fluctuations for expanding anti- and pro- pages (i.e. with an increased fan count) over non-expanding ones (i.e. with an unchanged or decreased fan count), we first use all features in table 1 in logistic regression (LR) to predict page status as either expansion or non-expansion. Furthermore, with the same goal in mind, we employ two supervised machine learning models: support vector regression (SVR) and random forest regression (RFR) to investigate the precise fan count variations within three categories of pages: all anti- and pro- pages, anti- and pro- pages that experienced expansion between February and October 2019, and anti- and pro- pages that remained non-expanded. Results are indicated in table 2. Our findings show that logistic regression moderately distinguishes between page expansion and non-expansion (accuracy: 0.655), with a stronger capability to identify expanding pages over non-expanding ones (sensitivity: 0.839; specificity: 0.153). This aligns with our initial expectation that recommendations can correlate more with fan size increase than decrease. Additionally, these results are supported by the notable performance improvements of SVR and RFR when specifying predictions exclusively for expanding pages, compared with predictions for non-expanding pages. For example, the *R*^2^ values of SVR and RFR on expanding pages are 0.11 and 0.17, which are much higher than those for non-expanding pages, −0.05 and −0.18, and those for all anti- and pro- pages, −0.02 and −0.02.

**Table 2.** Results about supervised machine learning (model performance). We apply parameter grid search and fivefold cross-validation on logistic regression (LR), support vector regression (SVR) and random forest regression (RFR). We note that the dataset in LR is unbalanced with 73.0% expanding pages encoded as ‘1’, and for this, we adjust the penalty weights for different classes in the cost function. Regarding the evaluation metrics, we optimize LR using accuracy as its metric, and we include sensitivity and specificity as metrics for further check. In our context, ‘sensitivity’ (also known as true positive rate) is explained as the proportion of expanding pages that are correctly classified as such, while ‘specificity’ (also known as true negative rate) is explained as the proportion of non-expanding pages that are correctly classified as such. We select mean absolute error (MAE) as our evaluation metric to optimize the performance of SVR and RFR, given its robustness to outliers in our dataset. We also provide *R*^2^ and root mean square error (RMSE) as additional metrics for validation. Regarding the baseline models, LR’s baseline model uses a random classifier in which each class is assigned an equal probability for prediction; SVR and RFR’s baseline models use the mean of fan count variations as their predictors.

				support vector regression (SVR)									random forest regression (RFR)								
	logistic regression (LR) (Expanding: 1; Non-expanding: 0)			all anti- and pro- pages			expanding anti- and pro- pages			non- expanding anti- and pro- pages			all anti- and pro-pages			expanding anti- and pro- pages			non-expanding anti- and pro-pages		
	accuracy	sensitivity	specificity	R^2^	MAE	RMSE	*R*^2^	MAE	RMSE	*R*^2^	MAE	RMSE	*R*^2^	MAE	RMSE	*R*^2^	MAE	RMSE	R^2^	MAE	RMSE
all features	0.655	0.839	0.153	−0.02	978.55	2854.72	0.11	1163.30	2884.36	−0.05	317.26	1088.53	−0.02	1084.06	2792.56	0.17	1127.20	2521.70	−0.18	352.83	1067.11
baseline	0.517	0.490	0.525	−0.02	1383.30	2808.18	−0.02	1697.50	3062.42	−1.11	554.28	1048.91	−0.02	1383.30	2808.18	−0.02	1697.50	3062.42	−1.11	554.28	1048.91

To evaluate the significance of each feature in our predictions above, we employ the correlation coefficient (CC) to measure linear dependencies between numeric features and fan count variations, again across three page categories (i.e. all anti- and pro- pages, expanding anti- and pro- pages, and non-expanding anti- and pro- pages). We also apply mutual information (MI) to capture nonlinear dependencies among all numeric and categorical features and fan count variations within these three page categories. Moreover, we employ sequential forward floating selection (SFFS) [43], a widely used feature selection method, to identify the optimal feature subset yielding the best performance in SVR and RFR models, exclusively for expanding anti- and pro- pages only. This decision is based on the better interpretability and performance of predictions for expanding pages, as previously confirmed. The results are presented in table 3. Our findings indicate that among categorical features, bow-tie relevant features (i.e. *W* − *BT*_*i*_ and *A* − *BT*_*i*_) exhibit relatively strong performance, with *A* − *BT*_*i*_ outperforming *W* − *BT*_*i*_. For example, the MI of *A* − *BT*_*i*_ regarding expanding anti- and pro- pages is notably higher than other categorical features. Also, SFFS reveals that both *W* − *BT*_*i*_ and *A* − *BT*_*i*_ significantly surpass polarity *p*_*i*_ and Infomap community *c*_*i*_ in the SVR model, although this advantage diminishes somewhat in the RFR model. Results about numeric features help explain the interpretability of our models. Of all numeric features, *f*_*i*_ (i.e. the page fan count in February 2019) exhibits the highest significance across all three page categories, as examined through CC, MI and SFFS. This observation, coupled with the opposite sign of its CC within expanding and non-expanding pages (0.485 versus −0.372), may indicate the ‘snowball effect’ [44], where the fan count of each page tends to either increasingly grow or decreasingly drop over time (i.e. pages with larger initial fan counts generally experience more substantial changes). Additionally, the higher significance of $k_{i}^{\mathrm{in}}$ compared with $k_{i}^{\mathrm{out}}$ is reasonable, with the former representing the strength of recommendations directed to each page and the latter indicating recommendations made by each page.

**Table 3.** Results about supervised machine learning (feature comparison). Owing to the stochastic nature of both mutual information (MI) and sequential forward floating selection (SFFS), we conduct 50 runs for both methods on expanding anti- and pro- pages and present the average MI value for each feature and the selected feature frequencies, respectively. Furthermore, the SFFS model identifies the optimal feature subset comprising 10 features to ensure that at least one categorical feature is selected in each run (i.e. for categorical feature comparison).

		all anti- and pro- pages		expanding anti- and pro- pages		non-expanding anti- and pro- pages		SFFS—using expanding anti- and pro- pages times chosen	
		CC	Avg. MI	CC	Avg. MI	CC	Avg. MI	SVR	RFR
categorical	*p*_*i*_	—	0.0011	—	0.	—	0.0714	9	48
	*c*_*i*_	—	0.0403	—	0.0002	—	0.1872	1	28
	*W* − *BT*_*i*_	—	0.0131	—	0.0001	—	0.0788	34	21
	*A* − *BT*_*i*_	—	0.0345	—	0.0424	—	0.0179	36	36
numeric	*f*_*i*_	0.379	0.3331	0.485	0.4277	−0.372	0.2884	50	50
	$k_{i}^{\mathrm{in}}$	0.406	0.1013	0.416	0.1501	−0.036	0.0770	50	41
	$k_{i}^{\mathrm{out}}$	0.254	0.0199	0.263	0.0360	−0.267	0.0012	50	42
	$k-PS_{i}^{\mathrm{in}}$	−0.039	0.0458	−0.107	0.0636	0.237	0.0033	40	29
	$k-PS_{i}^{\mathrm{out}}$	−0.078	0.0008	−0.137	0.	0.145	0.0432	49	50
	$k-CS_{i}^{\mathrm{in}}$	0.091	0.0088	0.053	0.0037	0.275	0.0117	49	39
	$k-CS_{i}^{\mathrm{out}}$	0.052	0.0228	0.044	0.0170	0.074	0.0052	39	37
	*PageRank*_*i*_	0.252	0.0958	0.258	0.1203	−0.032	0.5394	50	40
	*Betweenness*_*i*_	0.142	0.0170	0.127	0.0170	0.032	0.0008	43	38

Overall, supervised machine learning predictions of page fan count changes from February to October, based on the February online recommendation network, are generally more feasible and interpretable for expanding pages. Features related to bow-tie structure demonstrate potential in helping to predict the fan count variations of expanding pages.

#### 4.2.2. Agent-based susceptible-infected-recovered model

In our online recommendation-based networks, a recommendation from page A to page B is often triggered when page B shares content that captivates the interest of members of page A. This positive exchange of information may lead to page B acquiring new fans from page A. Motivated by this, we use an SIR model to simulate information cascades from each page and compare their information influence with fan count variations.

The SIR model, initially developed for simulating disease spread [45], has been used for modelling information diffusion in social networks [46–49]. In a network-based SIR model, each node can be in any of three states: susceptible (unaware of the circulating information), infectious (aware through creation or reception and willing to spread further), or recovered (aware but no longer transmitting, e.g. due to information obsolescence). Susceptible nodes can transition to an infectious state through contact with infected neighbours, the probability of which is proportional to the transmission rate *β*. Infectious nodes can transition to a recovered state spontaneously, with a probability of *γ*. A SIR epidemic process is usually initialized with one randomly selected node *i* being infected and terminated when no infectious nodes remain, simulating the propagation of a singular piece of information [46].

In our dataset, we run the agent-based SIR model on the February 2019 recommendation-based network. Consider to this end a total of *N* pieces of information generated during the period from February to October (for comparison with the October 2019 snapshot). For each individual piece of information *n* ∈ {1, 2, …, *N*}, the initial page that generates it, denoted as $I_{n}^{0}$, is selected randomly from a pool of anti- and pro- pages, following the probability distribution $P^{\mathrm{initializer}}.$^5^ The set of pages that are impacted by information piece *n* (i.e. in a recovered state at the end of the SIR dynamics about information *n*) is represented as *I*_*n*_. The influence of information piece *n* is defined as the aggregate fan counts of the pages impacted by it, represented by $\sum_{\;j\in I_{n}}f_{j}$. Subsequently, the influence of a page *i* is defined as the accumulated influence of all information pieces originating from page *i*, represented by $\sum_{n=1}^{N}1_{I_{n}^{0}=i}(\sum_{\;j\in I_{n}}f_{j})$. In order to account for how such information spread relates to the bow-tie components, we further divide the influence of each information piece into two categories: within-group and across-group. The former accounts for the influence of information piece *n* initialized from a pro- or an anti- page to pages with the same vaccination stance, and the latter accounts for the influence of information piece *n* from a pro- or an anti- page to neutral pages. Note that we disregard the influence of information piece *n* from a pro- page to an anti- page (or the other way around), as we assume users who follow pro- (anti-) pages may hardly accept information from anti- (pro-) pages and consequently follow them.^6^ We define the influence measures as follows:

$$Info\;W-Influ_{n}=\sum_{\;j\in I_{n}}1_{\;p_{I_{n}^{0}}=p_{j}}f_{j},\;p_{I_{n}^{0}}\in \{r,b\}$$

and

$$Info\;A-Influ_{n}=\sum_{\;j\in I_{n}}1_{\;p_{j}=g}f_{j},\;p_{I_{n}^{0}}\in \{r,b\}.$$

Subsequently, the within-group and across-group influence of a pro- or an anti- page *i* are defined below,

$$Page\;W-Influ_{i}=\sum_{n=1}^{N}1_{I_{n}^{0}=i}\left(\sum_{\;j\in I_{n}}1_{\;p_{i}=p_{j}}f_{j}\right),\;I_{n}^{0}∼P^{\mathrm{initializer}}$$

and

$$Page\;A-Influ_{i}=\sum_{n=1}^{N}1_{I_{n}^{0}=i}\left(\sum_{\;j\in I_{n}}1_{\;p_{j}=g}f_{j}\right),\;I_{n}^{0}∼P^{\mathrm{initializer}}.$$

Such within-group and across-group influences establish connections with within-group and across-group bow-tie components, respectively. This enables our following analysis, where we explore the potential influence distinctions among various bow-tie components, both within-group and across-group, and investigate whether these distinctions can aid in extracting pages’ roles in terms of information flow (i.e. SCC, ‘magnifiers’; OUT, ‘creators’; IN, ‘listeners’) and predicting variations in fan counts for each page.

**Figure 4.**
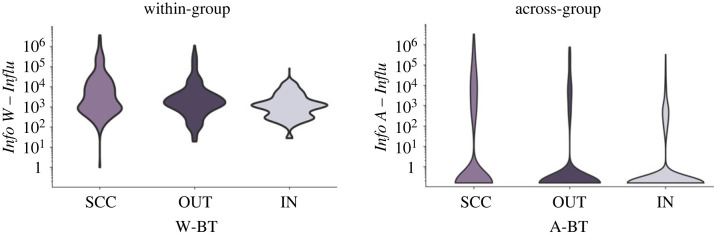
Distinctions among the influence of information pieces initialized from different bow-tie components. Each bow-tie component generates 1000 information pieces, with each page inside a component holding an equal probability of being the initial source for a single information piece, and the SIR epidemic process for each information piece is set at *β* = 0.5 and *γ* = 0.3. The violin plots depict the distribution of information within-group (across-group) influence, based on initialization from different within-group (across-group) bow-tie components. We observe that the hierarchy of influence, both within-group and across-group, adheres to the following ranking: SCC > OUT > IN. This aligns with the roles of these components (i.e. SCC, ‘magnifiers’; OUT, ‘creators’; IN, ‘listeners’) formed by the bow-tie decomposition. Additionally, a large quantity of information pieces remain confined within-group and have limited dissemination across-group, mirroring the real-world dynamics.

We first examine disparities in the influence of information pieces (i.e. *Info W* − *Influ*_*n*_ and *Info A* − *Influ*_*n*_) originating from different bow-tie components. Our results in figure 4 show that the hierarchy of influence, both for within-group and across-group, adheres to the following ranking: SCC > OUT > IN. This aligns with the roles of these components (i.e. SCC, ‘magnifiers’; OUT, ‘creators’; IN, ‘listeners’). Also, a substantial quantity of information pieces remain confined within-group and have limited dissemination across-group, mirroring the real-world dynamics.

**Figure 5.**
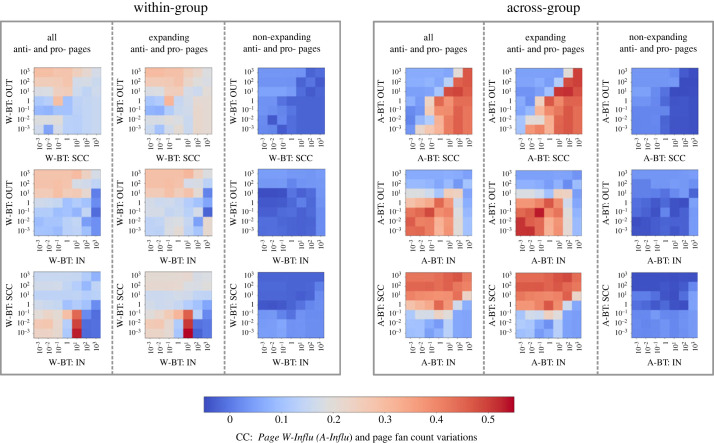
Distinctions among the influence of pages in different bow-tie components when varying their probability of generating information pieces. Each heatmap pixel represents the CC between the page influence and the page fan count fluctuations during the specified SIR epidemic process. We generate *N* = 3000 pieces of information for each pixel with *β* = 0.5 and *γ* = 0.3 (aligning with figure 4), and customize the probability of generating information $P^{\mathrm{initializer}}$ for pages in different bow-tie components. Specifically, in each heatmap, the *x*- and *y*-axes represent the information generation probabilities for pages in the respective bow-tie components, divided by the information generation probabilities for pages in other components that are not involved in either axis. Our results indicate that both within-group and across-group page influence appear more correlated with fan count variations of expanding pages, in contrast to non-expanding pages. The higher CCs of across-group page influence than within-group potentially suggest that the increase in fan counts for anti- and pro- pages may be more strongly influenced by their interactions with neutral pages instead of similar-minded ones. In the light of these two points, the upper-left red corner in the *W-BT:OUT* and *W-BT:SCC* heatmap illustrates that within-group OUT pages (‘creators’) are likely to produce more information pieces that recruit fans of pages sharing the same vaccination stance, compared with SCC pages. This result is also supported by other within-group heatmaps. Conversely, across-group SCC pages (‘magnifiers’) tend to generate more information pieces that possibly recruit neutral pages’ fans compared with OUT pages. Both across-group and within-group IN pages (‘listeners’) exhibit a limited trend in generating information pieces contributing to fan size variations, though surprisingly having a good performance when *x* = 10 in the heatmap related to *W-BT:SCC* and *W-BT:IN*. Moreover, the CCs of within-group and across-group page influence can maintain relatively high values compared with other numeric features in table 3. This demonstrates their high potential in aiding the prediction of fan count increases for anti- and pro- pages.

Secondly, we explore the disparities among the influence of pages (i.e. *Page W* − *Influ*_*i*_ and *Page A* − *Influ*_*i*_) in different bow-tie components, to investigate whether pages in certain bow-tie components tend to generate more information pieces that contribute to their fan count variations, by adjusting $P^{\mathrm{initializer}}$. We use CC as the metric to measure the association between page influence and fan count variations, as $P^{\mathrm{initializer}}$ varies.^7^ Our results are shown in figure 5. As we can observe, both within-group and across-group page influence appear more correlated with fan count variations of expanding pages, in contrast to non-expanding pages, aligned with our expectation (i.e. recommendations correlate more with fan size increase than decrease) and our ML results above. The higher CCs of across-group page influence than within-group potentially suggest that the increase in fan counts for anti- and pro- pages may be more strongly influenced by their interactions with neutral pages instead of similar-minded ones. This again aligns with our previous results in ML models (i.e. *W* − *BT*_*i*_ versus *A* − *BT*_*i*_). In the light of these two points, the skewed CC heatmaps in our results illustrate that within-group OUT pages are likely to produce more information pieces that recruit fans of pages sharing the same vaccination stance, compared with SCC pages. Conversely, across-group SCC pages tend to generate more information pieces that possibly recruit neutral pages’ fans compared with OUT pages. This finding helps explain a possible scenario, where OUT pages (‘creators’) often generate ‘innovative’ content that captures the interest of like-minded users (within-group), potentially inspiring content in SCC pages, while SCC pages (‘magnifiers’) amplify certain ‘mature’ content across-group, targeting neutral pages. Both across-group and within-group IN pages (‘listeners’) exhibit a limited trend in generating information pieces contributing to fan size variations, though surprisingly having a good performance when *x* = 10 in the heatmap related to *W-BT:SCC* and *W-BT:IN*. Notably, compared with the CCs between fan count variations of expanding anti- and pro- pages and other numeric features in table 3 (e.g. February fan count *f*_*i*_: 0.485; *PageRank*_*i*_: 0.258), the ones of our within-group page influence can maintain around 0.25, and across-group page influence around 0.5, demonstrating high potential in helping predict fan count increase for anti- and pro- pages.

Finally, the results above remain robust across different parameter choices for transmission rate *β* and recovery rate *γ*. See details in the electronic supplementary material.

## 5. Conclusion and future work

In this paper, we investigate bow-tie structure of discursive communities (i.e. groups of users sharing common communicative purposes) in temporal online social networks that describe the recommendations between anti-vaccination, pro-vaccination and neutral pages, with snapshots taken in February and October 2019.

By employing dual interpretations of discursive communities (one as vaccination groups and the other as communities detected by Infomap), we perform bow-tie analyses of recommendation networks within and across vaccination groups. Our results indicate different bow-tie structures among various vaccination groups. In both bow-tie analyses, a large number of anti-vaccination pages are assigned OUT bow-tie roles, while a substantial portion of pro-vaccination pages are assigned SCC bow-tie roles. These bow-tie structures exhibit statistical significance and demonstrate stability over the considered time frame. By contrast, the neutral group displays different bow-tie structures across these two analyses and demonstrates less temporal stability than the pro- and the anti- group.

We then relate these detected bow-tie structure differences to opinion dynamics, investigating their potential to predict changes in page fan counts from February to October using the February network. We implement both supervised machine learning models involving a variety of features, and mechanistic models on information cascades focusing on explainability, with these two approaches complementing and validating each other’s results. All our models are more adept at predicting page expansion (i.e. an increase in fan count) over non-expansion (aligning with our expectations), and bow-tie structure features exhibit promise in enhancing the prediction for expanding pages. Notably, such promise is indicated both in the performance of our models (e.g. bow-tie features *W* − *BT*_*i*_ and *A* − *BT*_*i*_ significantly surpass polarity *p*_*i*_ and Infomap community *c*_*i*_ in our SFFS–SVR machine learning model), and in the high interpretability of our agent-based models. For example, in our mechanistic simulation model, within-group OUT bow-tie pages—‘creators’—are shown to produce more information pieces that possibly recruit fans of pages sharing the same vaccination stance, while across-group SCC bow-tie pages—‘magnifiers’—tend to generate more information pieces that possibly recruit neutral pages’ fans.

### 5.1. Future work

There are a number of interesting directions to explore in future work. Firstly, the large OUT and SCC bow-tie components detected in the anti- and pro-vaccination groups, suggest distinct advantages held by the anti- and pro-vaccination groups: the former exhibits a strong commitment to generating information, while the latter possesses a strong capability for information dissemination. Based on our findings, which indicate that bow-tie structures can aid in predicting increases in their fan counts at the page level, it would be interesting to develop a bow-tie-based model for a longer time-scale fan count prediction of anti- and pro-vaccination pages spanning decades, contrasting it with Johnson *et al*.’s model [13], which disregards page recommendations in the dataset.

Secondly, for this bow-tie-based long-term fan count prediction model mentioned above, it would be interesting to incorporate the temporality of bow-tie structures, in light of the stronger temporal stability observed in the anti- and pro-vaccination groups compared with the neutral group in our dataset. More broadly, this framework can be expanded into a generative model that can be used to produce synthetic networks with different levels of bow-tie role adherence (e.g. SCC, ‘magnifiers’; OUT, ‘creators’; IN, ‘listeners’). These synthetic networks can serve as benchmarks and aids for inferring the structure of empirical networks of interest. We emphasize that our interest in role structure in information flow is not uncommon [22,50–52] (e.g. Beguerisse-Díaz *et al.* [50] capture five different roles in Twitter users, including ‘listeners’, ‘diversified listeners’, ‘references’, ‘engaged leaders’ and ‘mediators’), therefore developing such models can be important to understand the behavioural ecology in online social networks. Reference [53] can be a useful starting point for constructing the modelling framework.

Thirdly, the approaches considered in this paper are general and can be applied to a range of social networks. For instance, we can use bow-tie structure to investigate potential distinctions between misinformation and scientific information dissemination. A recent paper [16] highlights that a minority of accounts are responsible for the majority of the misinformation circulating on Twitter, a pattern highly pertinent to our bow-tie structure analysis. Bow-tie structure may also help explain the phenomenon of infodemic in misinformation circulation, which describes the situation where exposure to an abundance of information undermines people’s ability to discern disinformation, thus facilitating its dissemination [17,54]. More broadly, bow-tie structure analysis can yield insights about the structure and evolution of social networks, which we hope will be a helpful addition to those designing intervention efforts that aim to mitigate misinformation in social networks.

Fourthly, when examining the relationship between bow-tie structure and page fan count variations, we perform mechanistic simulations on information cascades. For our purpose, we use the agent-based SIR model in order to incorporate bow-tie-related factors. There are several other models that one could use. These include information dissemination models that incorporate additional factors that may be relevant to our research questions [55–57]. For instance, Ferraz de Arruda *et al*. [55] introduce a ‘forgetting mechanism’ affecting rumour lifespan and [56] incorporate ‘delays’ in information spreading.

In this paper, we study opinion dynamics through the lens of fan count change. The literature on opinion dynamics is broad and diverse, with models ranging from independent cascade to threshold models [58,59], widely studied in the social and computational sciences [60,61]. Usually, opinion diffusion models analyse the network dynamics at the individual node level, e.g. with agents changing their minds based on the majority of their influencers. Our approach is different, as we look at nodes with expanding volumes—our fan sizes—based on the polarity of selected influencers and their own size. This suggests a novel framework for understanding opinion dynamics, whose study is interesting in its own sake.

## Data Availability

Data and relevant code for this research work are stored in GitHub: https://github.com/YuetingH/BT_Vaccination_Views and have been archived within the Zenodo repository: https://zenodo.org/records/10513913. Supplementary material is available online [62].

## Footnotes

1 Further details on stance annotation can be found in [13,14]. Here, neutral pages ‘focus around vaccines or another topic (e.g. a school parent association that has become linked to the vaccine debate but for which the stance is still undecided)’, as introduced by Johnson *et al.* [13].

2 Follow-up research [14] provides an extended version of this dataset with an additional two snapshots—November 2019 and December 2020 (during the initial stage of the COVID-19 pandemic)—although without time-stamped fan size data. This limits our bow-tie analysis. Therefore, we focus only on the original dataset in [13] in our main paper and present some results of the extended dataset in the electronic supplementary material.

3 For any sets *A* and *B*, we define *A* − *B* as a set such that for any *i* ∈ *A* − *B*, *i* ∈ *A* and i∉B.

4 In the across-group bow-tie structure, pages within communities containing fewer than five nodes were labelled ‘UNASSIGNED’, as bow-tie structure of small communities may not be meaningful in our context. See details in §3.2.

5 Note that we prevent the initialization of information from neutral pages, as their information pieces are not our primary focus, in line with our previously mentioned assumption. That is, $P_{i}^{\mathrm{initializer}}\;:=P(I_{n}^{0}=i)=0,\text{ if }p_{i}=g$. As indicated in table 1, *p*_*i*_ ∈ {*r*, *b*, *g*} denotes the polarity of page *i* for anti-, pro-, neutral, respectively.

6 This assumption is also supported by the dataset, as evidenced by the sparse direct recommendations between anti- pages and pro- pages indicated in figure 2*c*, and the Infomap community detection result in figure 3.

7 In figure 5, each heatmap adjusts page generation probabilities $P^{\mathrm{initializer}}$ based on *x*- and *y*-axes values and associated bow-tie components (*BT*_*X*_ and *BT*_*Y*_). For each pixel (*x*, *y*) in the heatmap, we generate *N* information pieces, with each page *i* having a probability $P_{i}^{\mathrm{initializer}}\;:=P(I_{n}^{0}=i)\propto m_{i}$ , scaled to sum up to 1, where *m*_*i*_ = *x*, *y* if *i* ∈ *BT*_*X*_, *BT*_*Y*_ and *m*_*i*_ = 1 otherwise. In this sense, the *x*- and *y*-axes intuitively represent the information generation probabilities for pages in the respective bow-tie components, divided by the information generation probabilities for pages in other components that are not involved in either axis. Finally, the colour in each heatmap pixel represents the CC between the page influence and the page fan count fluctuations in the specified situation.
